# In vitro cancer models as an approach to identify targetable developmental phenotypes in cancer stem cells

**DOI:** 10.1007/s44164-023-00051-2

**Published:** 2023-05-12

**Authors:** Adrian Biddle

**Affiliations:** Blizard Institute, Queen Mary University of London, London, UK

**Keywords:** Cancer, Development, Stem cell, In vitro, Therapeutics

## Abstract

Cancer therapeutics are often highly toxic to the patient, and they often elicit rapid resistance in the tumour. Recent advances have suggested a potential new way in which we may improve on this, through two important concepts: (1) that multitudinous pathway alterations converge on a limited number of cancer cellular phenotypes, and (2) that these cancer cellular phenotypes depend on reactivation of developmental processes that are only minimally active in adult tissues. This provides a rationale for pursuing an approach of ‘drugging the phenotype’ focussed on targeting reactivated cellular processes from embryonic development. In this concepts paper, we cover these recent developments and their implications for the development of new cancer therapeutics that can avoid patient toxicity and acquired resistance. We then propose that in vitro tumour and developmental models can provide an experimental approach to identify and target the specific developmental processes at play, with a focus on the reactivation of developmental processes in the cancer stem cells that drive tumour progression and spread. Ultimately, the aim is to identify cellular processes that are specific to developmental phenotypes, are reactivated in cancer stem cells, and are essential to tumour progression. Therapeutically targeting these cellular processes could represent a new approach of ‘drugging the phenotype’ that treats the tumour whilst avoiding patient toxicity or the acquisition of therapeutic resistance.

## Section 1: Developmental cellular processes are a potential new class of cancer drug targets that may minimise both toxicity and resistance

Cancer drugs work through a plethora of different mechanisms, but the vast majority rely on the basic concept that a particular process (e.g. cell division, or immune tolerance) is more important to the cancer cells than to the normal tissues of the adult human body. Thus, with the correct dose, the drug can kill the cancer cells without causing unacceptable toxicity to the patient. However, this often goes awry. Chemotherapy drugs, designed to interfere with DNA replication or cell division, preferentially kill fast-growing populations of cells. This often causes debilitating (and sometimes fatal) toxicity to fast-growing normal tissues, including the haematopoietic system, skin, and colonic mucosa. Likewise, immunotherapies are designed to sensitise the immune system on the basis that mutated ‘neoantigens’ on the surface of cancer cells make them more susceptible to immune clearance [1]. However, this often results in serious autoimmune complications in patients [2]. Drugs targeted to specific signalling pathways offer an opportunity to target mechanisms that are more specific to cancer (through the concept of oncogenic addiction to a specific pathway). However, these drugs often show low efficacy (e.g. EGFR inhibitor in head and neck cancer) [3]. Even where there is a dramatic effect on the tumour and extension of patient survival, the focus on a single molecular pathway appears to invite rapid acquisition of resistance in the tumour cells through activation of alternate signalling pathways (e.g. BRaf inhibitor in melanoma, which brings dramatic initial results but quickly elicits resistance) [4].

**Figure 1:**
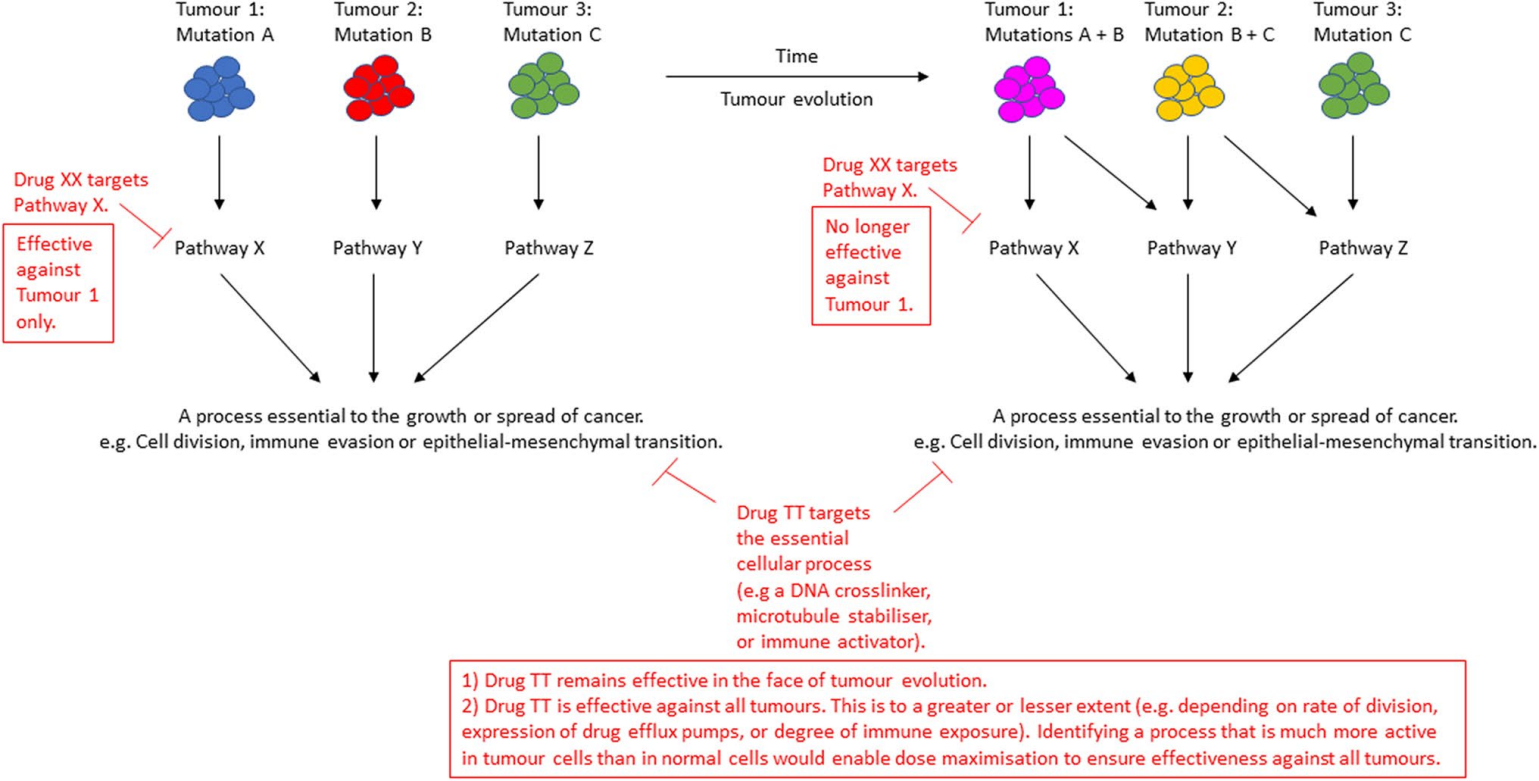
Drugging the phenotype. Convergence of many different genetic lesions and pathway alterations onto a much smaller number of essential cellular processes holds promise for developing a therapeutic approach that can limit the ability of cancer cells to develop resistance. Tumour evolution drives resistance to drugs targeting specific pathways (drug XX), but not to drugs targeting essential cellular processes (drug TT)

Thus, we are left with two options. We could focus on a broader range of specific signalling pathways through combination therapies designed to outfox the ability of tumour cells to acquire resistance through activating alternate pathways (much in the same way as combination antibiotic therapy for bacterial infections). Some promising early work has been performed on this topic, although it may require a personalised approach [5]. Alternatively, rather than focussing on individual signalling pathways, we could revisit the concept of drugging a broader cellular process. This has been the mainstay of cancer therapy, with the approaches of drugging cell division or immune tolerance described above, but dogged by high toxicity. However, if a cellular process could be found that is *much* more active in cancer cells than in the normal tissues of the human body, then this could present a way to drug the cancer whilst minimising associated toxicity. Importantly, convergence of many different genetic lesions and pathway alterations onto a much smaller number of essential cellular processes holds promise for developing a therapeutic approach that can limit the ability of cancer cells to develop resistance (Figure 1). By focussing on top-level cellular processes, this approach could be conceived as ‘drugging the phenotype’.

However, is it reasonable to suppose that there exist cellular processes that are much more active in cancer cells than in the whole gamut of specialised cells that make up the human body? Additionally, how might such cellular processes be identified? Two recent publications hint at a potential conceptual approach to solving these questions [6, 7]. In analysing human colorectal tumour specimens [7], the authors identified a disconnect between genetic lesions (that were predominantly clonal, that is shared between all cells in the tumour) and gene expression (extremely heterogeneous across the tumour, and evolving in response to microenvironmental stimuli). Additionally, gene expression profiles clustered into a discrete number of key patterns that were not genetically determined. This supports the emerging view that evolution of gene expression programs within a tumour can progress in the absence of concurrent genetic evolution, much in the same way as in a developing organism. Rather, the genetic complexity exhibited by tumours is largely dispensable to the evolution of their key gene expression patterns. By extension, if we posit each key gene expression pattern to represent a specific cellular phenotype, this suggests that genetic lesions affecting individual pathways may be somewhat redundant to the specification of cellular phenotypes within a tumour. Put another way, it supports the concept of convergence of many different genetic lesions and pathway alterations onto a much smaller number of essential cellular processes governing the observed phenotypes.

Further, the authors also show epigenetic changes associated with developmental plasticity in tumour development, with increased chromatin accessibility at binding sites for transcription factors involved in both embryonic development and epithelial-mesenchymal transition (EMT) [6]. This is in the absence of ongoing genetic evolution, and the epigenetic changes are stable and clonally inherited throughout the tumour, suggesting that they are essential to tumour development. Clonal genetic mutations in chromatin modifier genes correlated with this epigenetic evolution of the chromatin state. This suggests a scenario where driver mutations occurring early in tumour development initiate reprogramming of the tumour epigenome, which in turn re-activates a program of gene expression plasticity that is a feature of embryonic development and is essential for tumour development. This reprogramming includes activating the process of EMT, which is known to be important in embryonic development. These findings suggest epigenetic reprogramming to developmental plasticity in tumour development, a concept that we previously proposed [8] but for which experimental evidence has until recently been lacking (with the exception of melanoma and glioblastoma, where an understanding of the associated developmental stem cell phenotypes has enabled some progress on this front; reviewed in [9] and [10]).

Thus, from these two papers, we now have firmer evidence supporting two important concepts: (1) that multitudinous pathway alterations converge on a limited number of cancer cellular phenotypes, and (2) that these cancer cellular phenotypes depend on reactivation of developmental processes that may exhibit only minimal activity in adult tissues. This paves the way to an approach of ‘drugging the phenotype’ focussed on these reactivated processes from embryonic development.

In vitro experimental models have an important role to play in further testing and refining the key concepts that have come out of these studies on human tissue specimens. In particular, whilst human specimen studies provide important correlative evidence in actual human tumours, in vitro experiments can add a longitudinal and functional aspect that is able to go further and demonstrate causation. Whilst experimenting on primary tumour tissue in vitro is challenging and may introduce inappropriate selection pressures [11], for many tumour types there exists a good selection of established cell lines that accurately recapitulate the behaviour of the primary tumour of origin. One such tumour is oral squamous cell carcinoma (OSCC), for which there exists a range of well-characterised cell lines that demonstrate the same range of phenotypes and behaviours as the associated in vivo tumours [12–14]. This enables the selection of a panel of cell lines exhibiting the same genetic and phenotypic heterogeneity as in vivo human tumours, which in future can be used to experimentally test the two key concepts emerging from the human tissue studies. This in vitro approach can also be used to identify the developmental phenotypes required for key events in cancer progression, such as the local invasion and metastasis via the regional lymph nodes that are the main cause of OSCC patient death [15]. Specific developmental processes associated with these phenotypes may then present novel therapeutic targets. In the next section, we will discuss how we might use in vitro cell line experimental models to achieve this.

## Section 2: Using in vitro cancer models and the cancer stem cell concept to realise the promise of developmental cellular processes as cancer drug targets

If the acquisition of developmental cellular phenotypes were to represent key bottlenecks in tumour development, for example being required for metastatic dissemination, then this could provide important new therapeutic targets. Compared to targeting a specific signalling pathway, these targets would be much more applicable across populations of genetically heterogeneous patient tumours. They would also present less opportunity for the development of resistance, as genetically acquired alterations in individual pathways would be redundant to the requirement for the identified cellular phenotype in tumour progression. Indeed, there is evidence to support the existence of reactivated developmental phenotypes as crucial mediators of tumour progression. This comes from the cancer stem cell (CSC) field, where there is an appreciation that CSCs are both the phenotypic output of reprogramming to developmental plasticity in tumours [8] *and* crucial mediators of tumour progression [16].

CSCs therefore represent a developmental cellular phenotype that could be drugged to stop tumour progression. However, it is now well-established that CSCs are themselves highly phenotypically heterogeneous; the reactivation of developmental plasticity in CSCs enables them to adopt diverse cellular processes and phenotypic manifestations in order to drive tumour progression and spread, and survive insults from both hostile tumour environments and therapeutic interventions [12, 17–19]. These same attributes, driven by cellular plasticity, likely also enable successful development of the early embryo [20]. Given CSC plasticity and consequent diversity of cellular processes, there is now a challenge to determine which CSC attributes represent key developmental processes that are both dispensable to adult tissue and essential to the tumour.

A range of cellular processes have been identified as important to CSCs. These include EMT [21–23], autophagy [24], endoplasmic reticulum stress response [12], fatty acid metabolism [25], exosome release [26], and integrin-mediated matrix interactions [27], amongst others. However, many of these likely also play a role in normal adult tissue homeostasis, and as such may invite therapeutic toxicity in the same way as current chemotherapy drugs. Instead, focussing on plasticity as a cellular process *in itself* may provide a more fruitful approach to identifying druggable developmental targets that are specific to CSCs. Developmental cellular plasticity (as opposed to adult multi-lineage differentiation potential; see later) is notably absent from the homeostatic maintenance of normal adult tissue, despite sometimes being reactivated upon injury [28, 29].

Several of the cellular processes identified above as being important to CSCs have also been implicated in plasticity [12, 30, 31]. Plasticity may also rely on the ability to enact rapid change, suggesting a potential role for methods of translation control including P-bodies and nucleolar remodelling [32], which haven’t yet been investigated in CSCs. The important distinction is to identify those processes that are *specific* developmental regulators of plasticity and have no essential function in adult tissue.

In order to identify specific developmental regulators that are essential to CSCs, we will need experimental models that enable the identification and analysis of (1) CSC cellular phenotypes that are essential to tumour progression, and (2) developmental cellular phenotypes that are comparable to these CSC cellular phenotypes and are specifically expressed only in development. Much progress has been made in pursuing aim 1, including the identification of EMT as an important driver of metastasis using both in vitro and in vivo experimental models. This includes the use of sophisticated 3D in vitro models, which we have reviewed elsewhere [33]. There has however been a focus on the signalling pathways that maintain these CSC phenotypes, including developmental signalling pathways [34], rather than the broader cellular processes that are essential to the phenotype.

**Figure 2:**
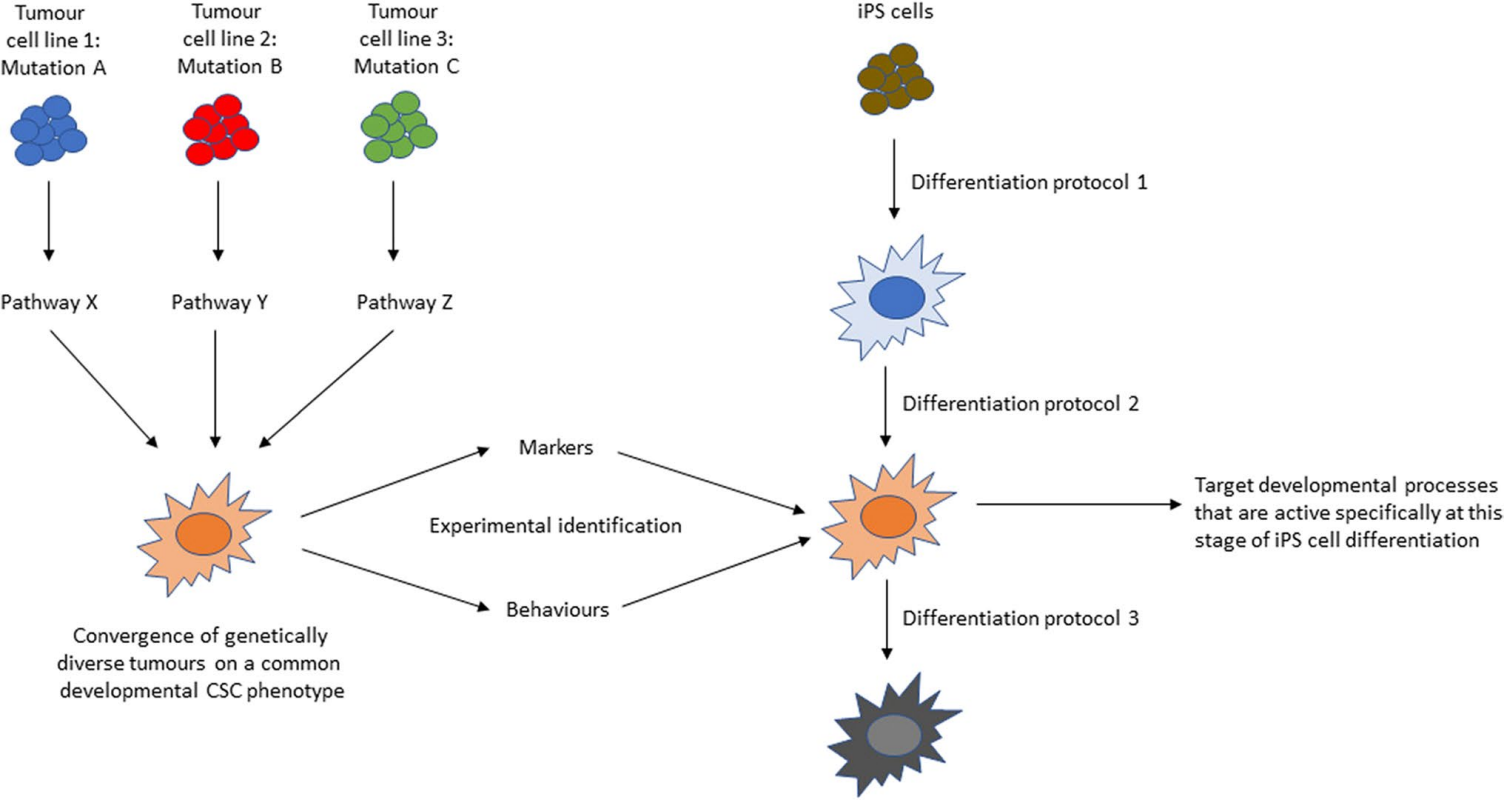
The potential for in vitro human models to identify developmental processes that are essential to the cancer stem cells that drive tumour progression. Differentiation of iPS cells through different developmental stages (right) can generate cellular phenotypes that can be compared to cancer stem cells (left) on the basis of their behaviours and stem cell marker expression in in vitro models. Once the correct cellular phenotype is identified, this can be assessed for essential cellular processes that are active specifically at this developmental stage, so that these can be targeted in cancer stem cells

Much less progress has been made in the identification of developmental cellular phenotypes, as required for aim 2 in the preceding paragraph (with the exception of those relating to melanoma and glioblastoma; reviewed in [9] and [10]). There have been a few studies of developmental trajectories in mice [35], that may serve as a starting point for identification of candidate phenotypes, but there is a lack of appropriate experimental models for identification of human developmental phenotypes. The ready availability of human iPS cells [36], pluripotent cells that can recapitulate developmental trajectories in vitro, provides a basis for the future development of in vitro human models that can be used to generate and characterise transitions through different developmental phenotypes, and identify which of these are analogous to CSCs (Figure 2). For example, CSCs in OSCC have been shown to co-express the epithelial marker EpCAM and the mesenchymal marker vimentin alongside the stem cell markers CD44 and CD24 [22]. Therefore, we may look for a developmental phenotype that expresses this same combination of markers. 3D in vitro models, including microfluidic chip models, can then demonstrate which cellular processes are essential to the developmental behaviour of these cells and how this may be relevant to CSC behaviours that drive tumour progression.

It will also be important to carefully delineate developmental cellular plasticity from the multi-lineage differentiation potential required for the maintenance of many adult tissues, most notably the blood [37]. Differentiation of iPS cells within in vitro model systems can be used to mimic human organ establishment and maintenance [38], again providing an experimental basis through which this comparison can be pursued.

Ultimately, the aim would be to identify cellular processes that are specific to developmental cellular phenotypes, are reactivated in CSCs, and are essential to tumour progression. Therapeutically targeting these processes could pave the way to a new approach of ‘drugging the phenotype’ that treats the tumour whilst avoiding patient toxicity or the acquisition of therapeutic resistance.
